# Radiographic Sagittal Alignment and Neurological Changes Following Conservative Cervical Structural Rehabilitation After Motor Vehicle Collision in a Patient With Pre-existing Scoliosis: A Case Report

**DOI:** 10.7759/cureus.104584

**Published:** 2026-03-02

**Authors:** Justin M Dick, Paris Paige

**Affiliations:** 1 Physical Medicine and Rehabilitation, Clear Life Scoliosis and Chiropractic Center, Charlotte, USA; 2 Chiropractic, Life University, Atlanta, USA

**Keywords:** cervical spine kyphosis, cervical whiplash syndrome, ligamentous cervical instability, motor vehicle accident (mva), neurological deficit, scoliosis management

## Abstract

Motor vehicle collisions (MVCs) are a frequent cause of cervical spine injury and may result in persistent neurological and structural sequelae in a subset of patients. Many individuals may improve with standard active management. Complex presentations involving sagittal malalignment and suspected segmental instability remain clinically challenging. The relationship between cervical alignment, dynamic motion behavior, and neurological status continues to be explored within contemporary spinal biomechanics literature.

This case report describes a 28-year-old woman who presented with persistent neck pain, neurological deficits, and functional limitation following a motor vehicle collision. The patient had a history of moderate thoracic scoliosis. Initial examination revealed forward head posture, reduced cervical extension, right-sided C8 sensory disturbance, and right C5-C8 motor weakness. Radiographic analysis demonstrated reduced cervical lordosis and abnormal segmental angular and translational motion on flexion and extension imaging. The Functional Rating Index (FRI) score at baseline was 23, indicating substantial disability.

The patient underwent a structured course of conservative spinal rehabilitation emphasizing sagittal alignment restoration, neuromuscular retraining, and segmental motion control over 17 visits. Follow-up evaluation at three months demonstrated normalization of dermatomal sensation and motor strength, improved cervical range of motion, and a reduction in functional disability with the Functional Rating Index decreasing to 3. Repeat radiographs showed increased cervical lordosis and decreased abnormal intersegmental translation.

This case documents concurrent neurological and radiographic changes in a post-collision presentation occurring in the context of pre-existing scoliosis. Although causation cannot be established from a single case, the findings highlight the potential relevance of evaluating cervical alignment and dynamic motion behavior in patients with neurologically involved whiplash presentations.

## Introduction

Motor vehicle collisions (MVCs) commonly injure the cervical spine by exposing it to rapid acceleration-deceleration forces that can stress osteoligamentous and neuromuscular stabilizing systems. Clinical presentations range from axial neck pain with restricted range of motion to more complex cases with objective neurological findings. These may include dermatomal sensory disturbance, motor weakness, and reflex asymmetry [1]. Many patients improve with early mobilization and graded activity consistent with whiplash management guidelines. A clinically meaningful subset develops persistent or complex symptom profiles despite these interventions [1,2]. Chronicity following whiplash-associated disorders (WAD) occurs in a substantial number of patients. This emphasizes the importance of identifying higher-risk phenotypes [2].

Structural factors, including altered cervical alignment and dynamic intersegmental instability, have been discussed as potential contributors to ongoing nociceptive and neurogenic mechanisms. Sagittal cervical alignment helps distribute load across the anterior column and posterior tension band while maintaining horizontal gaze [3,4]. Reduction of lordosis or progression toward kyphosis alters bending moments and stress transmission across discs, facets, and ligamentous structures [3]. Contemporary deformity metrics, such as the C2-C7 Cobb angle, C2-C7 sagittal vertical axis, and T1 slope-cervical lordosis mismatch, reflect a recognized association between sagittal imbalance and diminished health-related quality of life [4,5].

Sagittal malalignment has also been implicated in altered neural tissue mechanics. Cadaveric investigation demonstrates that increasing cervical kyphosis is associated with elevated spinal cord intramedullary pressure [6]. Finite element analyses similarly suggest that kyphotic alignment increases baseline spinal cord stress and strain. These effects may be amplified during flexion and extension, particularly in the presence of compressive forces [7]. Such findings offer mechanistic plausibility for considering sagittal configuration in neurologically involved presentations.

Rear-impact MVC biomechanics are heterogeneous and influenced by occupant posture and restraint geometry [8]. Experimental work indicates that whiplash-type loading may increase capsular ligament laxity and reduce ligament strength after trauma exposure [9,10]. Cadaveric models show that facet capsule strain during simulated rear-impact conditions can approach injury thresholds [11]. Collectively, the data supports a plausible structural substrate for altered segmental motion after injury.

Instability is a functional concept rather than solely an imaging diagnosis. This has been defined as failure of a motion segment to maintain normal intervertebral relationships under physiological load without producing neurological deficit or deformity progression [12]. Classical radiographic screening criteria describe translation exceeding 3.5 mm or intervertebral angulation greater than 11 degrees on flexion-extension radiographs as suggestive of instability [13]. In routine trauma pathways, computed tomography is typically prioritized for fracture exclusion. Flexion-extension radiography may be variably applied, potentially contributing to underrecognition of dynamic abnormalities [14].

Neurological involvement after whiplash further complicates management. Systematic synthesis indicates that neuropathic pain features and objective nerve pathology occur in a meaningful subset of patients with WAD [15]. Randomized clinical trial evidence demonstrates that neck-specific exercise may improve neurological signs and symptoms in chronic WAD populations [16]. However, most conservative intervention studies do not quantify sagittal alignment change or instability resolution as mechanistic endpoints.

Structural rehabilitation strategies targeting cervical lordosis have been evaluated in controlled trials. A systematic review of cervical extension traction methods reported measurable increases in lordosis associated with improvements in pain and disability across selected populations [17]. At the same time, meta-analytic and observational evidence demonstrate substantial variability in cervical curvature among asymptomatic individuals and an inconsistent association between lordosis magnitude and neck pain [18,19]. These findings caution against overly simplistic curvature symptom narratives.

Pre-existing scoliosis introduces an additional biomechanical context. Retrospective cross-sectional evidence demonstrates abnormal cervical intersegmental mechanics in patients with scoliosis [20]. This indicates that baseline structural asymmetry may influence cervical motion behavior independent of trauma. Integrated models of chronic whiplash emphasize interactions among biological injury, neurophysiology, and contextual factors [21]. Clinical practice guidelines continue to emphasize active management and exercise as foundational strategies [22]. Reciprocal alignment studies demonstrate that cervical posture adapts to global spinal balance. This suggests that pre-existing coronal asymmetry may influence cervical compensation patterns following trauma [23].

The present case describes a patient with post-MVC neurological deficits, cervical kyphosis, and clinical indicators suggestive of ligamentous instability in the setting of pre-existing scoliosis. Serial evaluation over three months demonstrated neurological normalization, improved range of motion, increased cervical lordosis, and a reduction in abnormal dynamic motion. This report aims to describe these concurrent changes within a unified biomechanical framework.

## Case presentation

Subject history

The study was conducted at a private integrative spinal rehabilitation clinic located in Charlotte, North Carolina. The facility's focus is non-surgical spinal corrective care. The data collection period spanned from November 14, 2025, to December 18, 2025, with a total of 17 office visits.

The subject of the case was a 28-year-old woman presenting on November 14th, 2025, with persistent and debilitating neck pain, fatigue, and low back pain following a motor vehicle collision on November 12, 2025. The subject denied previous similar traumas. The subject's spinal health history included a moderate thoracic scoliosis. She had not sought any other medical management pathways. The subject denied any form of topical or oral analgesics for the pain. The subject stated she was a full-time student and rated the pain 7/10 as a severe level of pain on a standard 0-10 numeric pain rating scale. Functional disability levels were assessed using the Functional Rating Index (FRI), administered at baseline, upon discharge, and at follow-up [24].

Physical examination and radiographs

Initial vital signs revealed a blood pressure of 99/70 mmHg. Heart rate measured 73 beats per minute. Postural examination revealed a forward head posture with a right low shoulder. Cervical range of motion was measured with PostureScreen (PostureCo, Trinity, FL) (Table 1). Aberrant motion and intersegmental dysfunction with associated loss of normal movement were identified throughout the cervical, thoracic, and lumbar spine. Palpation demonstrated tenderness within the cervical, thoracic, and lumbar paraspinal musculature bilaterally, with areas of increased muscle tone and spasticity noted across the left lumbopelvic region.

**Table 1:** Cervical digital range of motion Cervical range of motion was quantified using the PostureScreen® digital posture and movement analysis system. The software utilizes calibrated photographic capture and inclinometer input to objectively measure cervical flexion, extension, rotation, and lateral bending. Measurements were recorded in degrees (°) and used to assess asymmetry and motion restriction following lateral impact injury. AMA: American Medical Association, ARA: absolute rotational angle

Range of motion	AMA normal	Normal range	11/14/2025	2/12/2026	Finding 02/12/2026	Net change	Percent change
Right lateral flexion	45°	35°-50°	47°	49°	Within range	2	4%
Left lateral flexion	45°	35°-50°	47°	57°	Above range	10	21%
Cervical flexion	50°	40°-50°	66°	77°	Above range	11	17%
Cervical extension	60°	50°-70°	36°	40°	Below range	4	11%
Left rotation	80°	80°-90°	68°	83°	Within range	15	22%
Right rotation	80°	70°-90°	67°	92°	Above range	25	37%
ARA C2-C7 (segmental sum.)		-20° to -42°	19.1°	-	-	-	145.50%

Neurological assessment demonstrated objective findings consistent with the clinical presentation. These included altered sensorimotor responses and symptom provocation during targeted positional and functional testing. Neurological examination revealed right-sided sensory abnormality of the C8 distribution pattern. Associated motor deficits were present on the right with myotome strength, graded at 3/5 (N=5/5), across C5-C8. Sensory and motor testing on the left cervical was unremarkable (Table 2).

**Table 2:** Right-sided dermatomal and myotome findings on neurological examination Neurological examination revealed right-sided sensory abnormality of the C8 distribution pattern. Associated motor deficits were present on the right with myotome strength, graded at 3/5 (N=5/5), across C5-C8. Sensory and motor testing on the left cervical was unremarkable. The FRI is a composite measure derived from the Oswestry Low Back Disability Questionnaire and the Neck Disability Index. Scores range from 0 to 40, with higher scores indicating greater functional disability. The FRI has demonstrated strong reliability, validity, and responsiveness in clinical and research settings. FRI: Functional Rating Index

Spinal level	Dermatome right 11/14/2025	Myotome right 11/14/2025	Dermatome right 02/12/2026	Myotome right 02/12/2026
C1	Intact	Intact	Intact	Intact
C2	Intact	Intact	Intact	Intact
C3	Intact	Intact	Intact	Intact
C4	Intact	Intact	Intact	Intact
C5	Intact	3/5	Intact	Intact
C6	Intact	3/5	Intact	Intact
C7	Intact	3/5	Intact	Intact
C8	Positive	3/5	Intact	Intact
FRI	24	-	3	-

**Figure 1:**
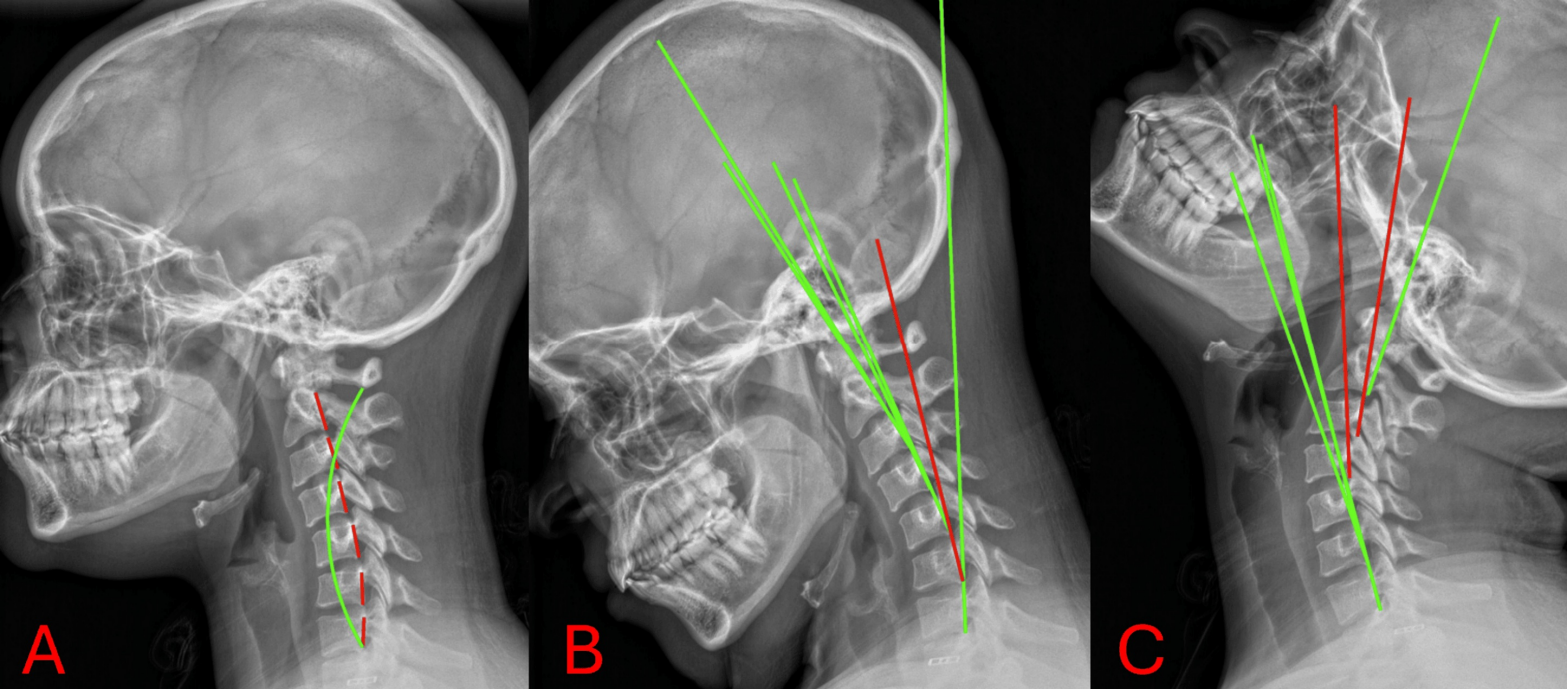
Lateral cervical neutral, lateral cervical flexion, and lateral cervical extension (11/14/2025) Panel A represents lateral cervical neutral. Panel B represents lateral cervical flexion. Panel C represents lateral cervical extension. The green reference line illustrates the expected posterior vertebral body alignment corresponding to the normal course of the posterior longitudinal ligament. The red reference line represents the subject's measured alignment using George's line, indicating deviation from ideal posterior vertebral body positioning. Posterior tangent lines were drawn along the posterior margins of the C2 through T1 vertebral bodies using the Harrison posterior tangent method. The green lines represent the theoretical ideal sagittal alignment model for comparison. Translational motion is measured by determining the anteroposterior motion of one vertebra over another vertebra. Angular loss of integrity is defined as a difference in angular motion between two adjacent motion segmental vertebra.

Radiographic evaluation demonstrated structural and alignment abnormalities that corresponded with both the physical and neurological examinations. Lateral neutral, flexion, and extension radiographs were acquired to review structure and function (Figure 1). Radiographic imaging was repeated following the completion of the care plan. Follow-up radiographs were obtained with identical positioning and exposure settings, and were performed and analyzed by the same physician to minimize variability. Quantitative cervical spine changes were measured to assess structural outcomes utilizing PostureRay (PostureCo, Inc., Trinity, FL), a machine learning-assisted parameter measurement. Prior studies have demonstrated high inter- and intra-examiner reliability of radiographic line-drawing methods with no significant differences compared to manual measurements across spinal regions. These metrics were compared with established normative data, as well as baseline imaging, to determine the degree of anatomical correction.

Lateral cervical radiographs revealed a persistent anterior head translation, decreased cervical lordosis, and abnormal segmental mechanics (Table 3).

**Table 3:** Flexion/extension measurements (11/14/2025) The ARA is a radiographic measurement method used to assess the total angle of the spinal curvature. Values in bold exceed the established normal of 11°. Values in bold exceed the established normal of 3.5 mm. ARA C2-C7 (global) refers to the absolute rotation angle measured across the entire cervical spine from C2 to C7 using posterior vertebral body tangents on a lateral radiograph. ARA: absolute rotation angle

Segment	Flexion ARA	Extension ARA	Angular excursion	Flexion translation	Extension translation	Translation excursion	
C2-C3	-1.4°	-10.3°	8.9°	0.3 mm	-2.1 mm	2.4 mm
C3-C4	7.5°	-10.9°	0.3°	0.2 mm	-4.0 mm	4.2 mm
C4-C5	1.8°	-11.8°	13.6°	0.3 mm	-2.1 mm	2.4 mm
C5-C6	9.7°	-5.1°	14.8°	0 mm	-0.7 mm	0.7 mm
C6-C7	11.8°	4.1°	7.7°	0.1 mm	-0.2 mm	0.3 mm
ARA C2-C7 (global)			N=-20° to -42°	19.1°	-	145.5% loss of curve	

Structural spinal correction was conducted using adjusting and neuromuscular retraining techniques derived from the CLEAR Institute protocols, which target spinal alignment restoration through their "mix, fix, and set" protocols of the CLEAR Institute [25].

**Figure 2:**
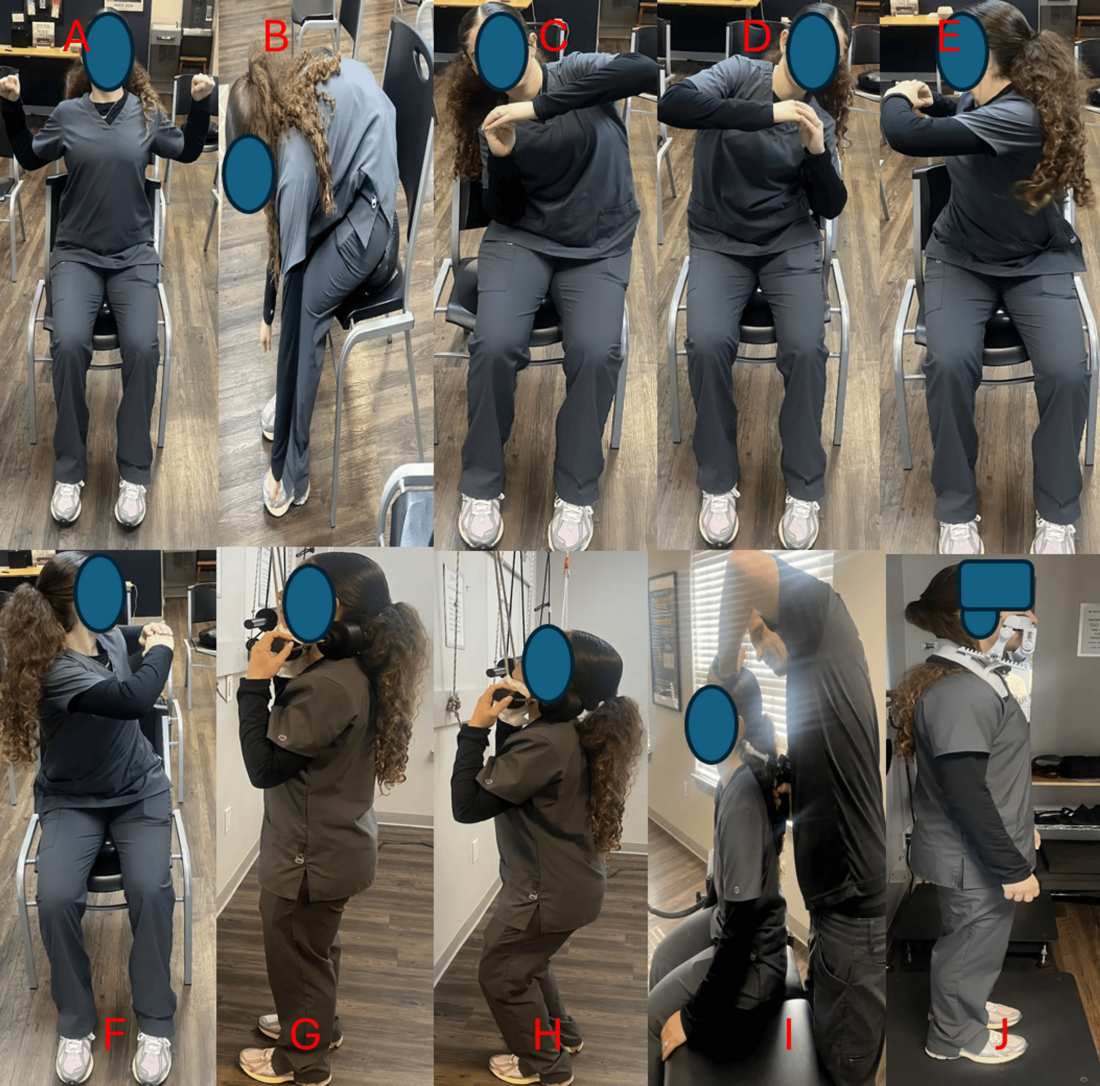
Therapeutic exercises and structural spinal rehabilitation Panels A-H demonstrate active rehab chair warm-up exercises (exercise therapy) to increase disc metabolism and flexibility through motion. Panel I demonstrates cervical chiropractic adjustments exclusively with an Arthrostim® (Arthrostim®, Salem, Oregon) due to abnormal cervical angulation and translation findings. Panel J demonstrates whole-body vibration with Cervigard (Cervigard Spinal Bracing, Inc., Bloomfield, NJ).

At each visit, treatment was delivered in a sequential manner consistent with the CLEAR Institute's "mix, fix, and set" framework. The total session duration was approximately 20 minutes. The initial phase consisted of active spinal mobility exercises intended to prepare soft tissues and facilitate segmental motion. The subsequent phase involved mechanical interventions directed at improving spinal alignment based on individualized radiographic findings. The final phase incorporated rehabilitative procedures designed to reinforce and stabilize the corrected alignment. Instrument-assisted or manual adjustments were performed at each visit in accordance with established CLEAR protocols. Whole-body vibration therapy was administered for approximately 10 minutes per session (Figure 2).

**Figure 3:**
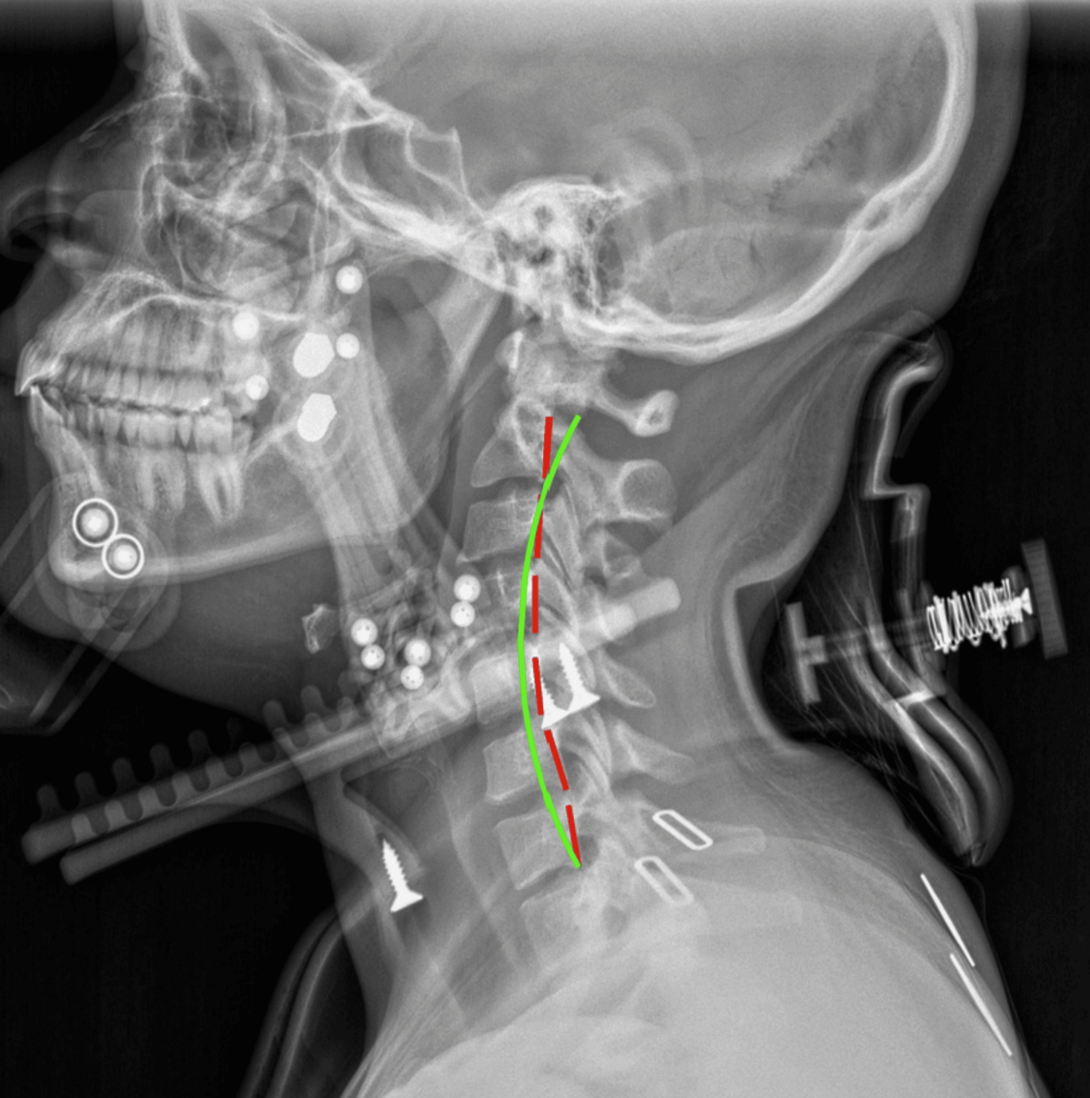
Stress radiograph (11/19/2025) The Cervigard orthotic is intended to reduce anterior head translation and promote restoration of cervical lordosis. This was prescribed to be worn 20 minutes daily during active treatment. ARA C2-C7 (global) was -14.2°. ARA measured across the entire cervical spine from C2 to C7 using posterior vertebral body tangents on a lateral radiograph (N=-20° to -40°). ARA: absolute rotation angle

The use of a Cervigard™ (Cervigard Spinal Bracing, Inc., Bloomfield, NJ) cervical orthotic was prescribed according to radiographic measurements (Figure 3). The Cervigard was used daily at home for 20 minutes in conjunction with the 17 office visits.

Follow-up evaluation

**Figure 4:**
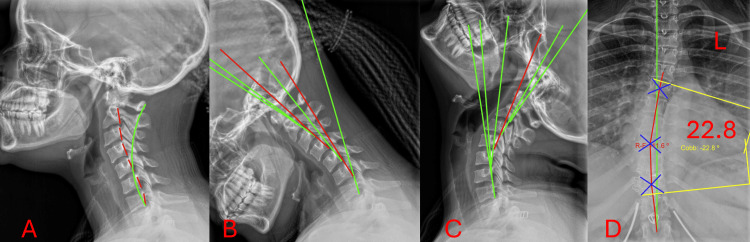
Lateral cervical neutral, lateral cervical flexion, and lateral cervical extension AP scoliosis radiograph (02/12/2026) Panel A represents lateral cervical neutral. Panel B represents lateral cervical flexion. Panel C represents lateral cervical extension. The green reference line illustrates the expected posterior vertebral body alignment corresponding to the normal course of the posterior longitudinal ligament. The red reference line represents the subject's measured alignment using George's line, indicating deviation from ideal posterior vertebral body positioning. Posterior tangent lines were drawn along the posterior margins of the C2 through T1 vertebral bodies using the Harrison posterior tangent method. Panel D represents an AP scoliosis radiograph. The green lines represent the theoretical ideal sagittal alignment model for comparison. Coronal radiographic analysis demonstrating scoliosis measurements. Yellow lines indicate Cobb angle measurements. Red lines depict the Risser-Ferguson analysis. Translational motion is measured by determining the anteroposterior motion of one vertebra over another vertebra. Angular loss of integrity is defined as a difference in angular motion between two adjacent motion segmental vertebra.

Follow-up evaluation on February 12, 2026, demonstrated cervical active range of motion improved in all planes and was no longer associated with pain at end range (Table 1). Repeat clinical examination demonstrated restoration of dermatomal sensory and manual muscle strength (Table 2). Repeat radiographic analysis was obtained using identical positioning and measurement protocols (Figure 4).

These demonstrated an increase in cervical lordosis (+44.3% (19.1° to 0.5° N=-20 to-42)) on neutral lateral imaging and reduced intersegmental translation on flexion and extension views (4.0 mm to 1.8 mm) compared with baseline. Flexion segmental RRA (C6-C7) changed from 11.8° and 11.8° on extension to 16.0°. Segmental RRA (C4-C5) changed from 11.8° extension to 15.7 RRA. Segmental angular excursion changed from 13.6° to 24.9° (Table 4). The Functional Rating Index improved from 23 at baseline to 3 at follow-up (Table 2). This reflects a reduction in functional disability and corresponding clinical recovery.

**Table 4:** Flexion/extension measurements (02/12/2026) The ARA is a radiographic measurement method used to assess the total angle of the spinal curvature. Values in bold exceed the established normal of 11°. Values in bold exceed the established normal of 3.5 mm. ARA: absolute rotation angle

Segment	Flexion ARA	Extension ARA	Angular excursion	Flexion translation	Extension translation	Translation excursion
C2-C3	3.3°	-5.2°	8.5°	0.3 mm	-2.1 mm	2.3 mm
C3-C4	6.1°	-7.2°	13.3°	0.2 mm	-1.8 mm	2.0 mm
C4-C5	9.2°	-15.7°	24.9°	1.8 mm	-1.7 mm	3.5 mm
C5-C6	6.2°	-11°	17.2°	1.6 mm	-0.8 mm	2.4 mm
C6-C7	16.0°	-4.3°	20.3°	0.0 mm	0.0 mm	0.0 mm
ARA C2-C7 (global)	-	-	N=-20° to -42°	0.5	-	101.2% loss of curve

## Discussion

This case illustrates a clinically complex post-MVC phenotype characterized by neurological deficits, reduced cervical mobility, radiographic kyphosis, and motion characteristics interpreted as suggestive of instability. Although uncomplicated post-collision neck pain frequently improves over time. A substantial number of patients develop persistent symptoms [2]. The coexistence of neurological findings and altered sagittal alignment raises questions regarding the interaction of mechanical and neurophysiological mechanisms in post-traumatic cervical presentations.

Meta-analytic evidence indicates that neuropathic pain characteristics and measurable nerve pathology are not rare following whiplash injury [15]. Against this background, the normalization of neurological findings in parallel with structural and motion changes warrants careful interpretation.

Biomechanical evidence supports the plausibility that kyphotic alignment may influence neural tissue mechanics. Increased kyphosis has been associated with elevated spinal cord intramedullary pressure in cadaveric models, and computational modeling suggests increased cord stress and strain in kyphotic configurations [6,7]. While these models cannot be directly extrapolated to individual patients, they provide a mechanistic basis for considering sagittal alignment as one potential modifier of neural irritability.

Similarly, ligamentous laxity following whiplash exposure has been demonstrated experimentally, and facet capsule strain during simulated loading approaches injury thresholds [9-11]. Instability defined by dynamic translation criteria may therefore represent a functional manifestation of altered segmental mechanics rather than a discrete pathological state [13]. Improvements observed over serial imaging in this case may reflect enhanced neuromuscular control, reduced abnormal loading, or natural recovery processes rather than structural ligament healing.

The observed increase in lordosis parallels findings from controlled studies examining extension-based rehabilitation approaches [17]. However, literature examining cervical curvature in asymptomatic populations demonstrates wide variability and inconsistent association with symptoms [18,19]. The novelty of this case lies not in asserting that lordosis predicts pain, but in documenting concurrent neurological, mobility, and alignment improvements within a post-traumatic phenotype where structural factors may have heightened relevance.

The integrated stress-diathesis model of chronic whiplash emphasizes dynamic interaction among biological injury, neurophysiology, and contextual factors [21]. The present findings are compatible with that framework and do not contradict active management principles [22]. Rather, they suggest that in patients with objective neurological deficits and measurable sagittal deformity, evaluation may benefit from inclusion of structural and dynamic metrics in addition to symptom scales.

Pre-existing scoliosis further contextualizes this case. Reciprocal alignment research demonstrates that cervical posture adapts to global spinal balance [23]. While largely derived from deformity surgery cohorts, these findings support the plausibility that coronal asymmetry may influence baseline mechanics and post-traumatic adaptation.

Many recent studies have documented measurable radiographic curve changes over time during conservative management [26,27]. The studies illustrate that longitudinal imaging can demonstrate structural change under monitored care. Complementary reporting examining the labyrinthine righting reflex further highlights that postural reflex mechanisms may influence structural alignment patterns [28]. These observations collectively reinforce the concept that spinal alignment and intersegmental behavior are dynamically influenced by neuromuscular control rather than representing fixed mechanical states.

Several limitations constrain interpretation. As a single case, spontaneous recovery cannot be excluded. Flexion-extension radiography carries inherent variability, and clinical interpretation of ligamentous instability lacks definitive confirmation in the absence of advanced imaging. Central sensitization and psychosocial influences, which are known contributors to chronic WAD, were not formally quantified.

Future research should focus on phenotype-specific investigation of post-MVC patients presenting with objective neurological signs. Prospective cohort studies that incorporate standardized neurological examination, validated patient-reported outcomes, and quantified sagittal and dynamic imaging metrics may provide greater clarity in this area. Such designs could help delineate how cervical alignment, segmental stability, and neural function interact over time. This case highlights the importance of considering cervical alignment, neurological findings, dynamic motion behavior, and pre-existing spinal morphology within a unified biomechanical framework following a motor vehicle collision. While definitive causal conclusions cannot be drawn, the multimodal coherence observed supports further structured investigation.

## Conclusions

This case documents parallel improvement in neurological function, cervical range of motion, sagittal alignment, and intersegmental motion following structural spinal conservative care in a patient with a history of motor vehicle collision and underlying scoliosis. Causal conclusions cannot be established from a single case observation. The temporal association between neurological recovery, radiographic modification, and functional improvement suggests that structural and dynamic factors may play a meaningful role in whiplash presentations with objective neurological findings. These observations support consideration of alignment and motion analysis as part of a comprehensive evaluation strategy. Future prospective studies should incorporate standardized neurological examinations, validated outcome measures, and quantified imaging parameters. Such designs are necessary to clarify the relationship between cervical alignment, segmental stability, and neural function after trauma.
